# Measuring Fidelity to Evidence-Based Practices: Psychometrics

**DOI:** 10.1007/s10488-020-01074-7

**Published:** 2020-07-31

**Authors:** T. Ruud, R. E. Drake, G. R. Bond

**Affiliations:** 1 Mental Health Services, Akershus University Hospital, Lørenskog, Norway; 2 Institute of Clinical Medicine, University of Oslo, Oslo, Norway; 3 Westat, Lebanon, NH USA

**Keywords:** Fidelity scales, Psychometrics, Evidence-based practices, Psychosis

## Abstract

This special section presents the psychometric properties of fidelity scales used in a national mental health services project in Norway to improve the quality of care of people with psychoses. Across Norway, 39 clinical units in six health trusts participated. The project provided education, implementation support and fidelity assessments. The papers in the section address the psychometrics of fidelity measurement for the specific evidence-based practices: illness management and recovery, family psychoeducation, physical healthcare and antipsychotic medication management. Another paper analyzes the psychometrics of a scale measuring individualization and quality improvement that may be used in conjunction with fidelity scales for specific evidence-based practices. The first paper in the section presents the development and field of fidelity scales, and the two final papers with comments add some additional perspectives and discuss fidelity scales in a wider context. The psychometrics of the five scales were good to excellent. Fidelity assessment is a necessary and effective strategy for quality improvement.

## Background for the Special Section on Fidelity Scales

Evidence-based practices and clinical guidelines can improve the quality of treatment in health care, including for people with psychoses. But accurate implementation of evidence-based practices and clinical guidelines in routine healthcare remains problematic. To achieve optimal outcomes, the field needs more effective strategies to assure faithful implementations.

Fidelity scales are tools to assess the quality of implementation in a program or clinical unit and guide improvements. “The rationale for using fidelity scales to guide practice is based on the working hypothesis that programs successfully replicating the core principles and procedures of the program models rigorously evaluated in controlled studies will achieve similar outcomes as these earlier studies” (Bond et al. 2009).

Widespread use of fidelity scales requires reliability and validity. The National Evidence-Based Practices (EBP) Project in the U.S. demonstrated a standardized method for development and format of fidelity scales (Bond et al. 2009) and methods for testing the properties for such scales (McHugo et al. 2007). Nonetheless, few studies of the psychometric properties of currently available fidelity scales exist. The papers in this special section address that deficit.

## The Context for Our Testing of Properties of Fidelity Scales

The testing of properties of fidelity scales in this section occurred as a part of a cluster randomized controlled trial on the implementation of four evidence-based practices for treatment of patients with psychoses in mental health services (ClinicalTrials NCT03271242). Thirty-nine sites (community mental health centers/inpatient departments) in six health trusts throughout Norway participated. The four practices—illness management and recovery, family psychoeducation, physical health care, antipsychotic medication management—are among the core evidence-based practices for treatment of people with psychosis in Norway.

Each site chose two of the four practices for implementation and agreed to receive implementation support for one of the two, determined by random assignment. The site received the following support for the practice randomized to implementation support: A workshop with experts at the start, a toolkit for implementing the practice, a visiting implementation trainer biweekly for 6 months and then monthly for 12 months, and telephone supervision for the two manualized psychosocial programs. Sites randomized to the control condition only received a written description of the practice. Two trained experts assessed fidelity of both practices at each site at baseline and after 6, 12, and 18 months using independent and consensus ratings. The papers in this special section report on psychometric properties of the fidelity scales for all practices at sites that received implementation support.

## Papers in the Special Section

The overview by Bond and Drake (2019) covers the history and current status of fidelity scales for evidence-based practices in mental health. It describes the use of fidelity scales, procedures for development and validation of fidelity scales, and benefits and challenges in using fidelity scales in research and in improvement of clinical programs and practice.

The following papers address the psychometric properties for each of the four fidelity scales and for the General Organizational Index (GOI). The scales for illness management and recovery and family psychoeducation are existing scales for well known, manualized psychosocial programs. The scales for physical health care and antipsychotic medication management are new scales measuring recommended practice components. The GOI is an established scale for measuring the overall organization of services. All five scales followed the guidelines for fidelity scale development described by Bond and Drake (2019).

Egeland et al. (2019) report on the Fidelity Scale for Illness Management and Recovery (IMR), a scale developed in the National EBP Project (McHugo et al. 2007) and subsequently used in several studies. The paper by Egeland et al. is the first comprehensive assessment of the psychometric properties of the scale. Joa et al. (2020) report on the Family Psychoeducation Fidelity Scale, also developed in the National EBP Project, and this paper is the first to report its psychometric properties.

Ruud et al. (2020a) report on the content and psychometric properties of the Physical Health Care Fidelity Scale for measuring a best practice approach to physical health care for people with psychosis. This is the first published fidelity scale to assess physical health care for people with psychosis. In a separate paper, Ruud et al. (2020b) report on the content and psychometric properties of the Antipsychotic Medication Management Fidelity Scale for measuring evidence-based recommendations for antipsychotic medication management.

Heiervang et al. (2020) report on the psychometric properties of the General Organizational Index (GOI), also developed in the National EBP Project. The GOI measures aspects of implementation common to all evidence-based practices but usually not included in fidelity scales for specific practices.

In his comments van Weeghel (2020) discusses some of the challenges and limitations in the fidelity scales reported in this section, overcoming resistance to fidelity measures, fidelity assessments in mental healthcare systems, and the importance of predictive validity. He also gives an international perspective by describing some developments in Europe, including in the Netherlands.

Wiltsey Stirman (2020) comments on challenges in the assessment of providers’ competence, observer-rated quality of service delivery and calibration of fidelity ratings. She emphasizes the need to assess the process of implementation in addition to fidelity assessments. She also discusses research on adaptions of interventions and distinguishes between core functions and forms of delivery of complex interventions that may be important in further development of both programs/interventions and of fidelity scales.

## Conclusions

The papers in this special section contribute to knowledge on psychometric properties of some fidelity scales for evidence-based practices in the treatment of people with psychoses. The section gives an overview and illustrates the present state of fidelity measurement and some aspects for further development and use of fidelity scales.
